# Supplementary material for: An ultrasensitive molybdenum-based double-heterojunction phototransistor
Source: Nat Commun. 2021 Jul 2;12:4094. doi: 10.1038/s41467-021-24397-x (PMC8253832; doi:10.1038/s41467-021-24397-x)
Supplement: Supplementary file 1 — Supplementary Information [file 41467_2021_24397_MOESM1_ESM.pdf]

Supplementary Information for

An ultrasensitive molybdenum-based double-heterojunction phototransistor

Feng *et al*.

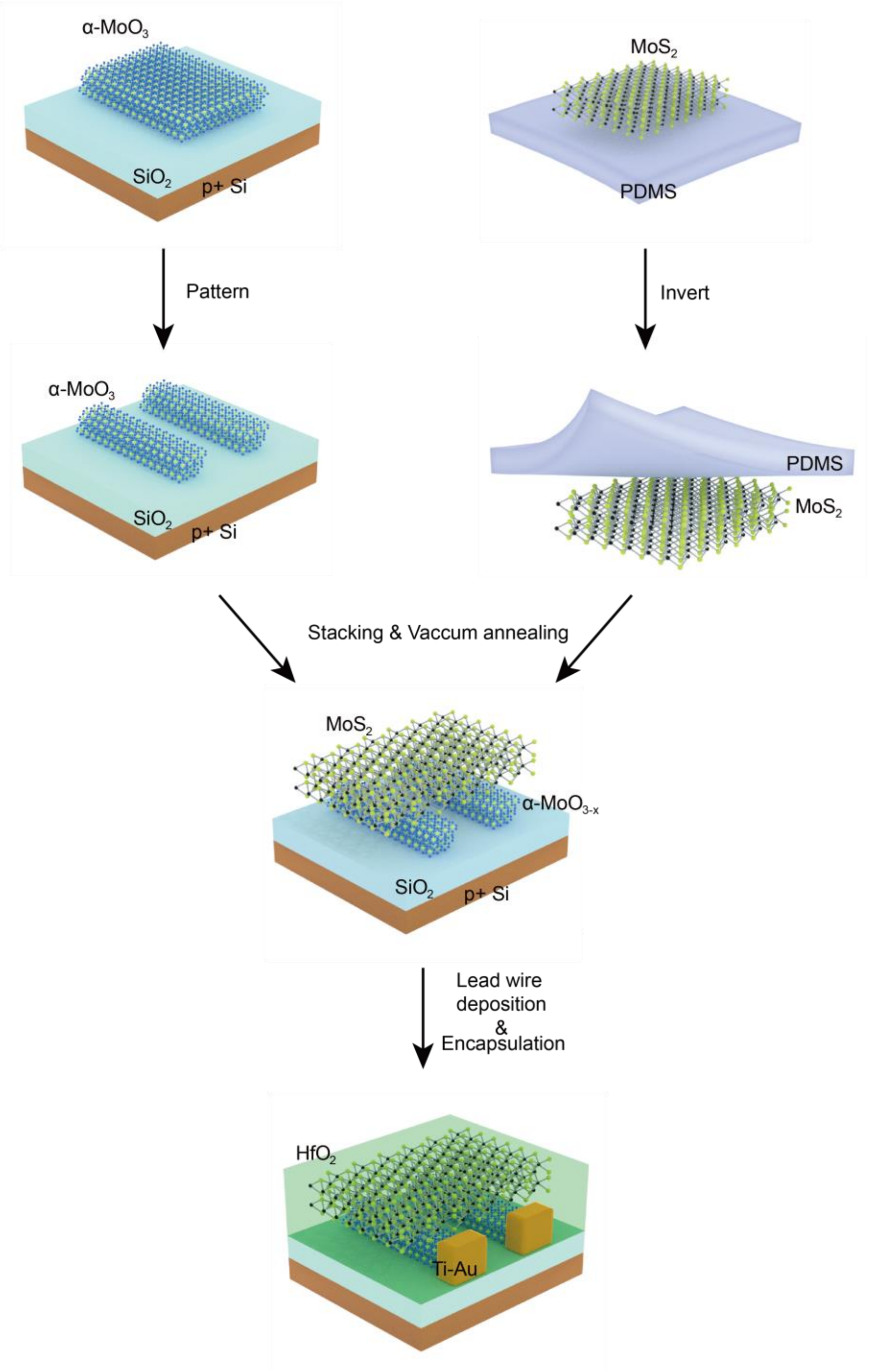

Supplementary Fig. 1 Device fabrication process. First, a multi-layer α-MoO_3 flake was exfoliated onto a SiO_2/Si substrate and patterned by RIE. A few-layer MoS_2 flake was exfoliated onto a PDMS substrate, and the few-layer MoS_2 flake was transferred onto the α-MoO_3 electrodes and the resulting stack was vacuum annealed. Metal contacts (Ti/Au: 5/50 nm) were then formed by EBL, electron-beam evaporation and lift-off processes. The device was finally passivated by a 5-nm-thick HfO_2 layer deposited by atomic layer deposition (ALD).

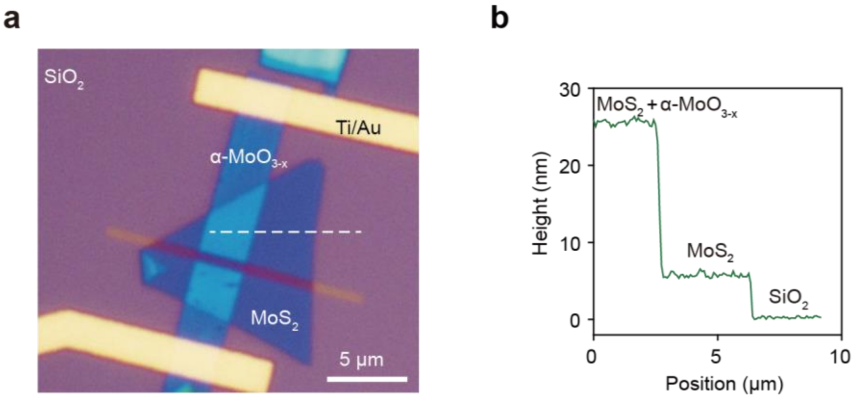

Supplementary Fig. 2 Thickness measurements. **a** An optical image of the α-MoO_{3-x}/MoS_2/α-MoO_{3-x} phototransistor. **b** Material thickness along the white dashed line in **a**, indicating a thickness of 5.4 nm and 20 nm for MoS_2 and α-MoO_{3-x}, respectively.

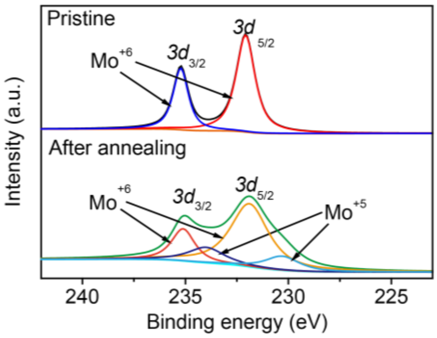

Supplementary Fig. 3 X-ray photoelectron spectroscopy (XPS) spectra of pure α-MoO_3 flakes and annealed α-MoO_{3-x} flakes for Mo *3d*. The Mo $3d_{5/2}$ and $3d_{3/2}$ peaks are located at binding energies of 232.07 eV and 235.19 eV and are assigned to the Mo^{6+} oxidation state of the α-MoO_3 phase[1-3]. After annealing, the Mo *3d* peaks broadened because of the appearance of an Mo^{5+} oxidation state. This result indicates that oxygen defects were introduced by annealing.

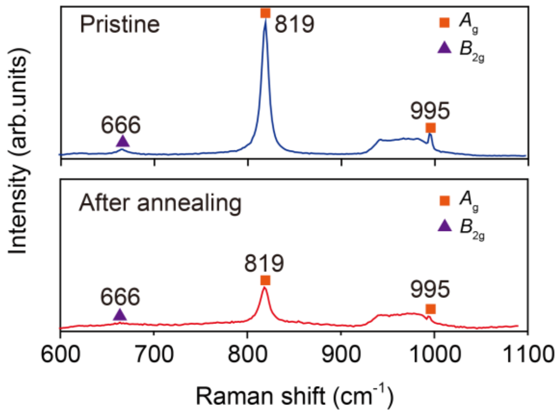

Supplementary Fig. 4 Raman spectra of a pure α-MoO_3 flake and the same one after annealing. The intensity of all three main peaks at Mo_3-O (666), Mo_2-O (819) and Mo_3-O (995) became weaker after annealing, suggesting that a some of the chemical bonds was broken.

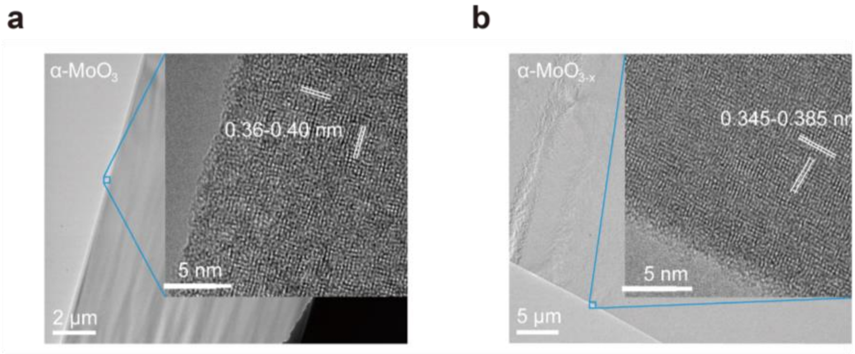

Supplementary Fig. 5 TEM characterization. **a** Pure α-MoO_3 flake showing a lattice constant of 0.36-0.40 nm. **b** Annealed α-MoO_{3-x} flake showing a lattice constant of 0.345-0.385 nm. The lattice constant of the crystal decreases slightly after annealing due to the presence of oxygen defects in the crystal.

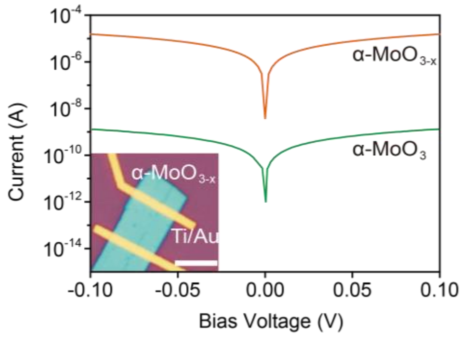

Supplementary Fig. 6 Electrical measurements of α-MoO_{3-x}. Dependence of the current on the bias voltage of α-MoO_3 and α-MoO_{3-x}. Inset: Optical image of an α-MoO_{3-x} device (scale bar: 5 μm). After annealing, the conductance of the α-MoO_{3-x} increases more than 4 orders of magnitude due to the doping of oxygen defects, consistent with previous reports[1-3].

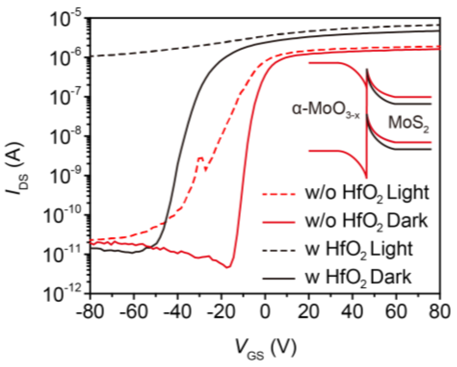

Supplementary Fig. 7 Optoelectronic performance of the α-MoO_{3-x}/MoS_2/α-MoO_{3-x} phototransistor with (w) and without (w/o) a HfO_2 doping layer. The transfer characteristics (I_{DS}-V_{GS}) of the phototransistor show that the threshold voltage shifts in the negative direction with the doping layer in the dark, indicating effective n-type doping of MoS_2. Inset: Such an n-type doped MoS_2 is necessary for obtaining an obvious photo response because it provides a sufficiently thin barrier in MoS_2 for electron injection from α-MoO_{3-x}.

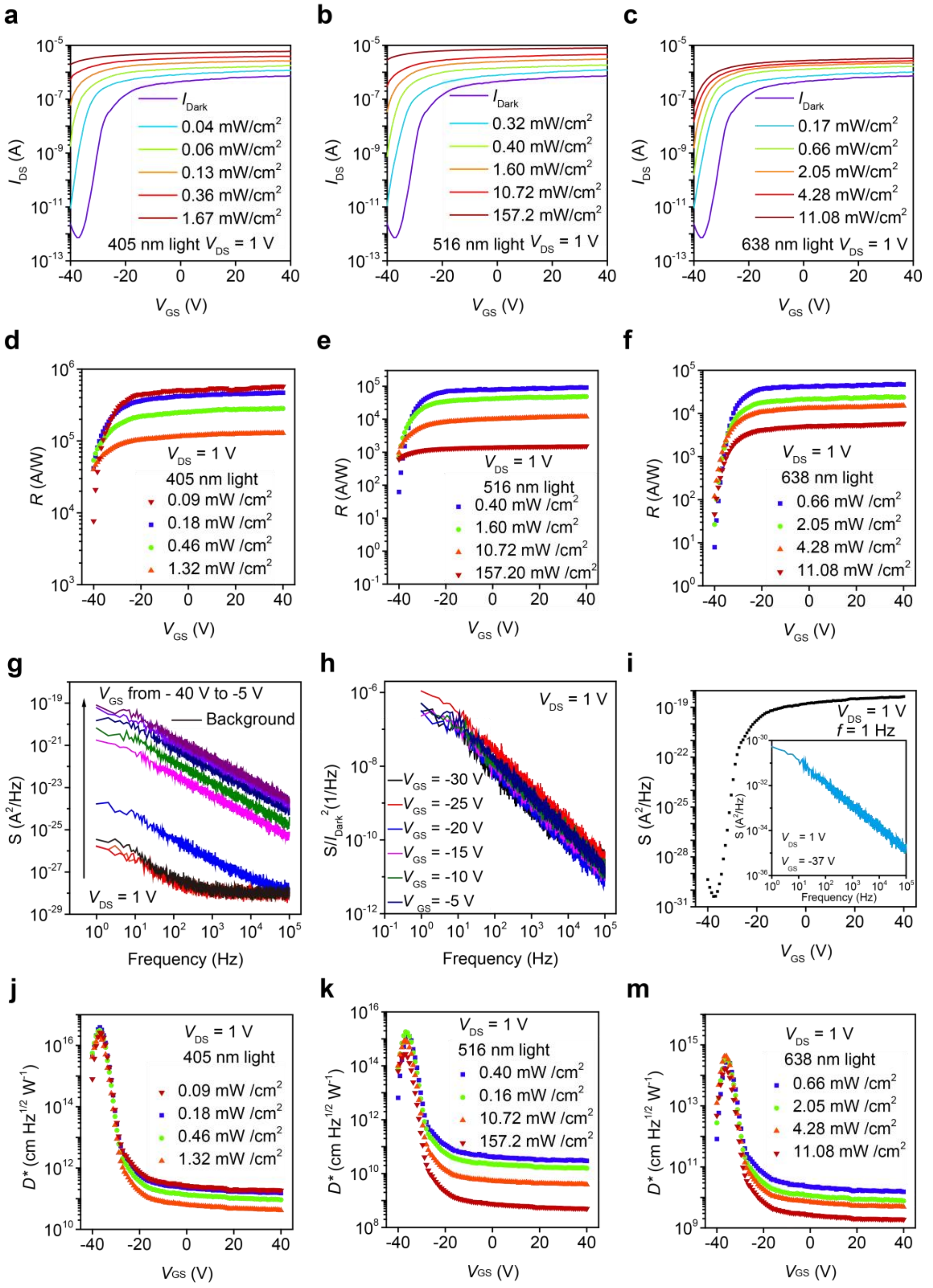

Supplementary Fig. 8 Optoelectronic performance of the phototransistor. The channel length and width of this device are 0.5 and 6 μm. **a** Transfer curves of this device at V_{DS} = 1 V in dark and under the 405 nm light. **b** Transfer curves of this device at V_{DS} = 1 V in dark and under the 516 nm light. **c** Transfer curves of this device at V_{DS} = 1 V in dark

and under the 638 nm light. **d** R as a function of V_{GS} with a 405 nm light at V_{DS} = 1V. **e** R as a function of V_{GS} with a 516 nm light at V_{DS} = 1V. **f** R as a function of V_{GS} with a 638 nm light at V_{DS} = 1V. **g** Noise power density (S) as a function of frequency at V_{DS} = 1 V with different V_{GS} of −5 V, −10 V, −15 V, −20 V, −25 V, −30 V, −35 V, −40 V, as well as the background noise (in black) from top to down. S decreases rapidly with a decrease of V_{GS}, and drowns with background noise when the V_{GS} decreases to be -35 V. **h** Normalized noise power density (S/I_{Dark}^2) as a function of frequency at V_{DS} = 1 V with different V_{GS} of −5 V, −10 V, −15 V, −20 V, −25 V and −30 V. **i** S as a function of V_{GS} at V_{DS} = 1V. Inset: S as a function of frequency at V_{DS} = 1 and V_{GS} = −37 V. **j** D^* as a function of V_{GS} with a 405 nm light at V_{DS} = 1V. **k** D^* as a function of V_{GS} with a 516 nm light at V_{DS} = 1V. **m** D^* as a function of V_{GS} with a 638 nm light at V_{DS} = 1V.

Supplementary Fig. 8g shows the relationship between current noise and frequency at various back gate voltages. All these low-noise spectra exhibit a typical 1/f power density. It is well-known that the 1/f (flicker) noise is mainly dominated by fluctuations of carrier density or mobility. The current I_{Dark} was extracted from the I_{DS}-V_{GS} characteristics in the dark (**Supplementary Fig. 8a**), and **Supplementary Fig. 8h** shows that the noise power spectral density S (f) is proportional to I_{Dark}^2, indicating that the photocurrent is not determined by external fluctuations such as interfacial traps[4].

As the normalized noise power density (S/I_{Dark}^2) is almost a certain value at different frequency (f), we can extract the real noise which drowns with background noise using $S = (S/I_{Dark}^2) \times I_{Dark}^2$. The S at different gate voltage at f = 1Hz is shown in **Supplementary Fig. 8i,** the real S (2×10^{-30} A^2/Hz) at V_{GS} = −35 V is far below the measured S (3×10^{-26} A^2/Hz). By this way, we also calculated the S at V_{GS} = −37 V under different f, shown in **Supplementary Fig. 8i Inset**. Here, we need to point out that the extracted S is not correct when the f is large at V_{GS} = −37 V, because the extracted S is

smaller than the shot noise (2e I_{Dark}) of this device.

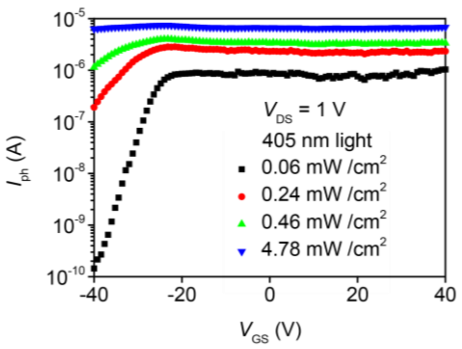

Supplementary Fig. 9 Photocurrent (I_{ph}) as a function of gate voltage V_{GS}. $I_{ph} = I_{DS} - I_{Dark}$.

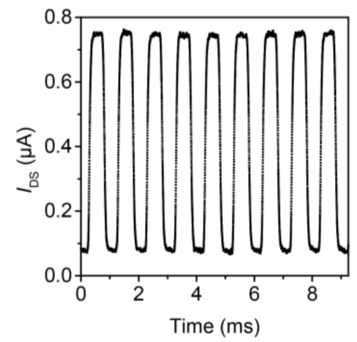

Supplementary Fig. 10 Response speed of α-MoO_{3-x}/MoS_2/α-MoO_{3-x} phototransistor. Photocurrent as function of time at bias voltage (V_{DS}) of 1 V and gate voltage (V_{GS}) of 0V with a modulation frequency of 1 kHz.

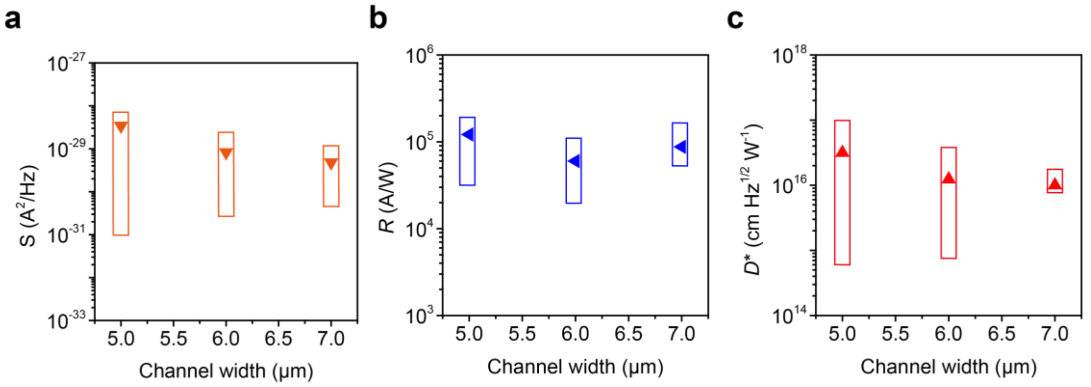

Supplementary Fig. 11 Statistical analysis of performance of phototransistors with different channel width using the 2-3 nm-thick MoS_2 and controlling the channel length about 0.5 μm. The *S*, *R* and *D** of our devices are fluctuated around 10^{-29} A^2/Hz, 10^5 A/W and 10^{16} cm Hz^2 W^{-1} respectively. The detailed data of these devices are shown in the **Table 1**. **a** Channel width dependence and statistics of *S*. **b** Channel width dependence and statistics of *R*. **c** Channel width dependence and statistics of *D**.

Number	Maximum of D^* (cm Hz$^{1/2}$ W^{-1})	V_{GS} (V)	R (A/W)	S (A^2/Hz)	Channel length (μm)	Channel width (μm)
1	9.8×10^{16}	-35.2	1.9×10^{5}	9.7×10^{-32}	0.5	5
2	2.1×10^{15}	-40.0	8.8×10^{4}	3.8×10^{-29}	0.5	5
3	5.9×10^{14}	-40.0	3.2×10^{4}	6.5×10^{-29}	0.5	5
4	3.6×10^{16}	-36.8	1.1×10^{5}	2.7×10^{-31}	0.5	6
5	1.2×10^{16}	-36.8	7.3×10^{4}	1.0×10^{-30}	0.5	6
6	7.1×10^{14}	-38.4	1.9×10^{4}	2.2×10^{-29}	0.5	6
7	8.6×10^{15}	-39.2	1.8×10^{5}	1.6×10^{-29}	0.5	7
8	1.6×10^{16}	-35.0	5.8×10^{4}	4.5×10^{-31}	0.5	7
9	7.3×10^{15}	-32.0	5.4×10^{4}	1.9×10^{-30}	0.5	7

Table 1 Summary of key performance parameters of nine devices.

In order to make the readers understand the differences between our devices, we determined the bias voltage (V_{DS} = 1 V), the range of gate voltage (changing from -40 V to 40 V) and the MoS_2 thickness (2-3 nm), extracted the maximum detectivity (D^*) of the device under 405nm laser, as well as the corresponding gate voltage (V_{GS}), responsivity (R), noise current density (S) and device size, shown in the **Table 1**. We provide detailed data of device Number. 1 in the main manuscript, and we also provide detailed data of device Number. 4 in the **Supplementary Fig. 8.**

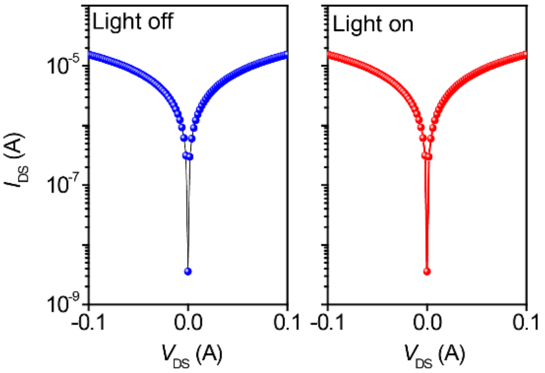

Supplementary Fig. 12 Photo response of α-MoO_{3-x}. We deposited two Ti/Au electrodes on the α-MoO_{3-x}. Then we test the I_{DS}-V_{DS} curves with a bias of 0.1 V in the dark (left) and under the light (right). Compared with the I_{DS}-V_{DS} curves in the dark, no photocurrent was generated in this device under a 405 nm laser with a power density of 1.8 mW/cm^2.

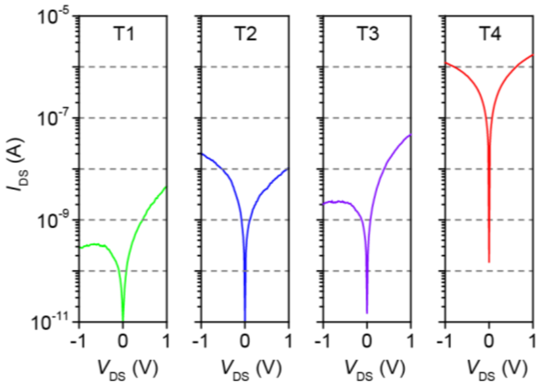

Supplementary Fig. 13 Output characteristics (I_{DS}-V_{DS}) of T1 to T4 measured using light with a wavelength of 405 nm and a power density of 0.2 mW/cm^2. V_{GS} = −80 V. Compared to a metal electrode (T1), I_{DS} increases for devices using one heterojunction as cathode (T2, T3), and most significantly when using double heterojunctions (T4).

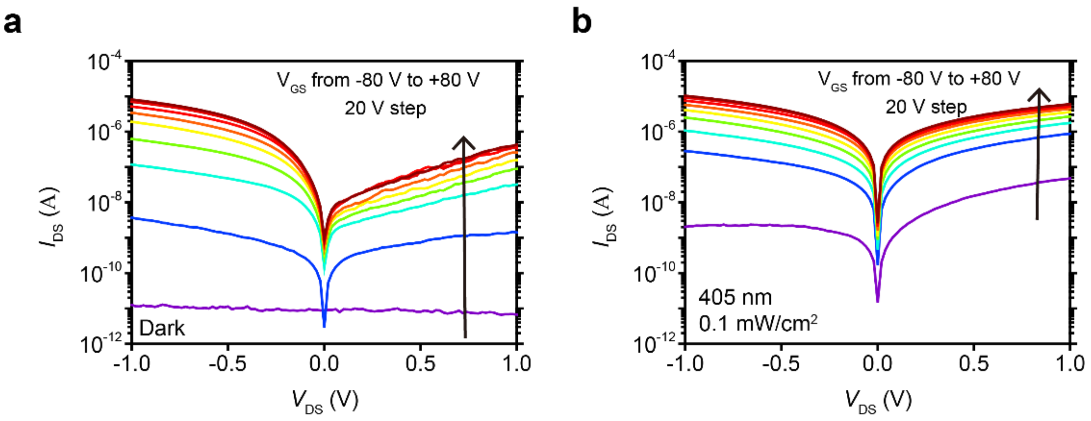

Supplementary Fig. 14 Photo response of the asymmetric device α-MoO_{3-x}/MoS_2/Ti-Au. **a** I_{DS}-V_{DS} characteristics of the asymmetric device in the dark with changing V_{GS} from −80 V to 80 V. **b** I_{DS}-V_{DS} characteristics of the asymmetric device under the light with changing V_{GS} from −80 V to 80 V.

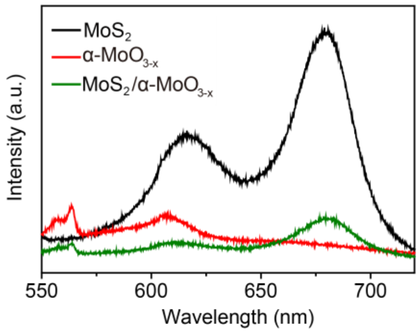

Supplementary Fig. 15 PL spectrum of MoO_{3-x}, MoS_2 and α-MoO_{3-x}/MoS_2 heterojunction.

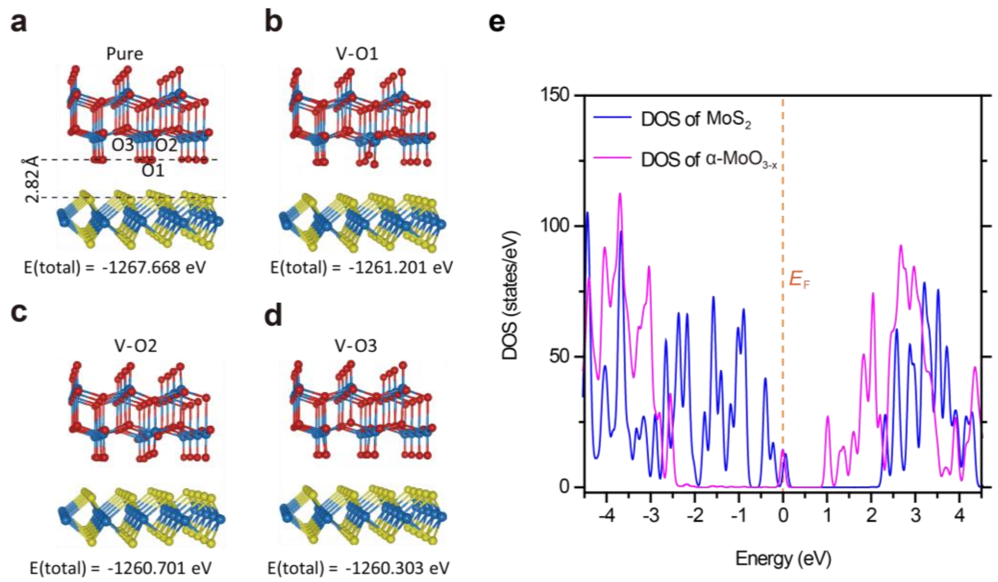

Supplementary Fig. 16 Schematic structures of an α-MoO_3/MoS_2 heterojunction consisting of **a** pure α-MoO_3, **b**, **c**, **d** α-MoO_{3-x} with oxygen vacancies produced by removing the O atoms labelled in **a**. O, Mo, and S atoms are denoted by red, blue, and yellow balls, respectively. The calculated DFT total energies (in eV) are also given to evaluate the structural stability. **e** Calculated density of states (DOS) for α-MoO_{3-x}/MoS_2 heterojunctions. The Fermi level was set to be 0 eV. The solid blue, purple, and dotted orange lines represent the DOS of MoS_2, the DOS of α-MoO_{3-x} and the Fermi level, respectively.

As confirmed by XPS, Raman and TEM characterization (Supplementary Figs. 3-5), the pure α-MoO_3 has changed to α-MoO_{3-x} due to the existence of oxygen vacancies after annealing. Similar to previous theoretical work[6-7], our DFT calculations show that oxygen vacancy formation by removing the O1 atom is most energetically favorable.

It should be noted that the difference between the E_c values for MoS_2 and α-MoO_{3-x} was calculated to be 1.3 eV (Fig. 4a), very close to the value of 1.1 eV obtained by experiment. However, the difference between the E_v values for MoS_2 and α-MoO_{3-x} was calculated to be 2.5 eV and is smaller than the experimental value (3.1 eV). This is due to that fact that monolayer MoS_2 with a large band gap value of about 2.0 eV was

used in the DFT calculations, while few-layer MoS_2 was used in the experiments.

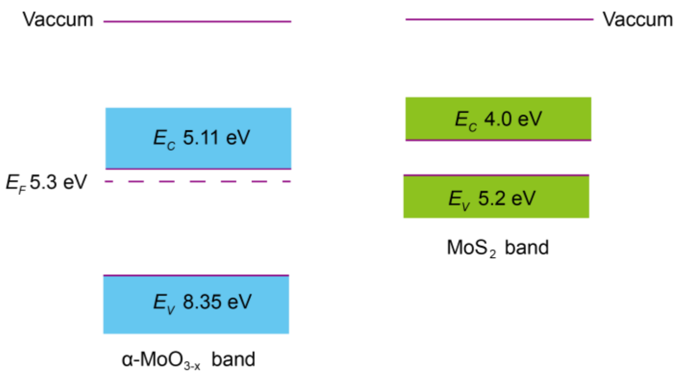

Supplementary Fig. 17 Band structure of α-MoO_{3-x} and MoS_2. The band structure of α-MoO_{3-x} was drawn from the absorbance spectrum, the UPS spectra and PL spectrum. E_C is the conduction band minimum, E_T is the defect band and E_V is the valence band maximum. The band structure of MoS_2 is from the literature[5].

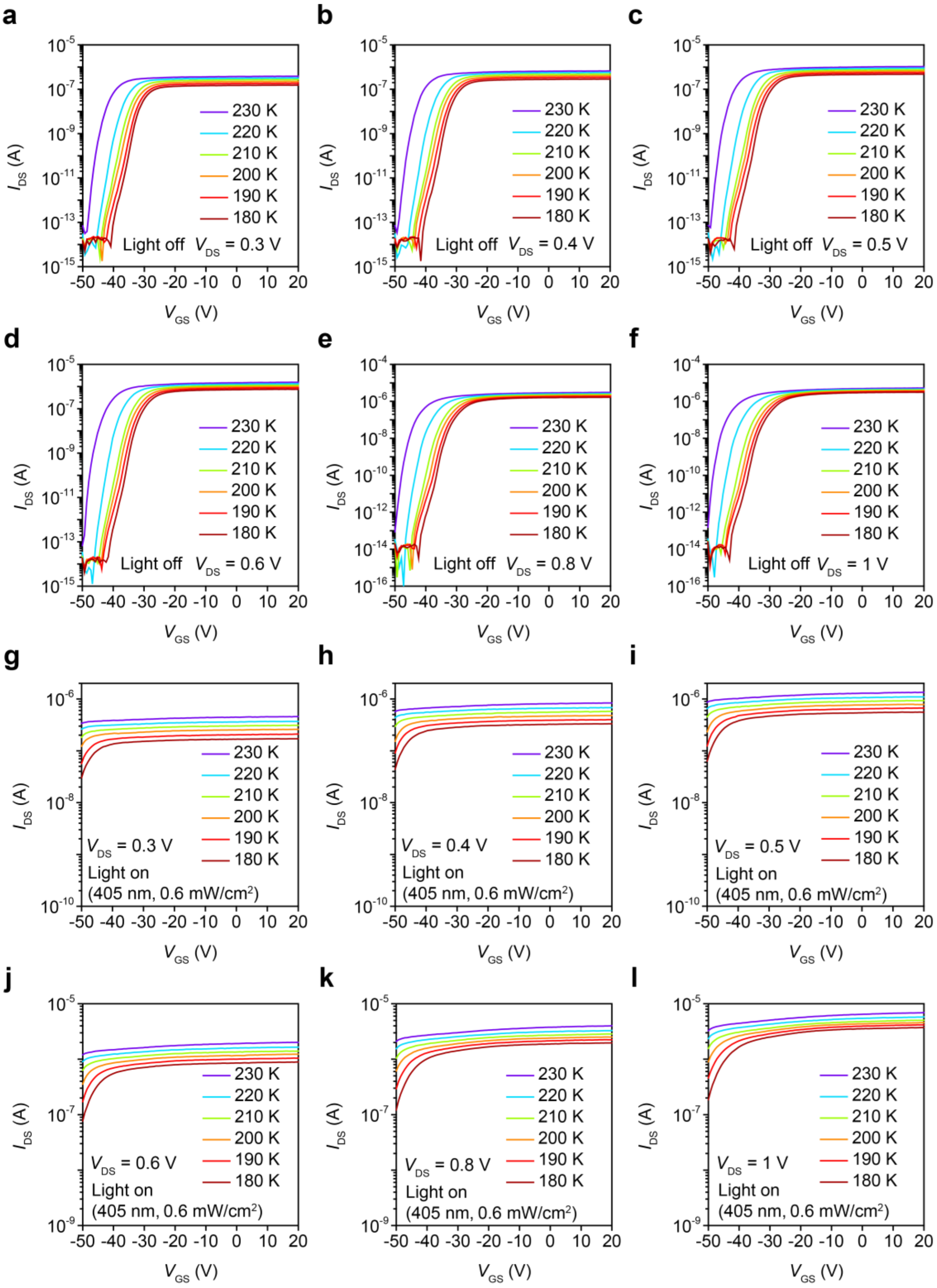
a
Light off V_{DS} = 0.3 V
b
Light off V_{DS} = 0.4 V
c
Light off V_{DS} = 0.5 V
d
Light off V_{DS} = 0.6 V
e
Light off V_{DS} = 0.8 V
f
Light off V_{DS} = 1 V
g
V_{DS} = 0.3 V
Light on
(405 nm, 0.6 mW/cm^2)
h
V_{DS} = 0.4 V
Light on
(405 nm, 0.6 mW/cm^2)
i
V_{DS} = 0.5 V
Light on
(405 nm, 0.6 mW/cm^2)
j
V_{DS} = 0.6 V
Light on
(405 nm, 0.6 mW/cm^2)
k
V_{DS} = 0.8 V
Light on
(405 nm, 0.6 mW/cm^2)
l
V_{DS} = 1 V
Light on
(405 nm, 0.6 mW/cm^2)
230 K
220 K
210 K
200 K
190 K
180 K
I_{DS} (A)
V_{GS} (V)
-50 -40 -30 -20 -10 0 10 20

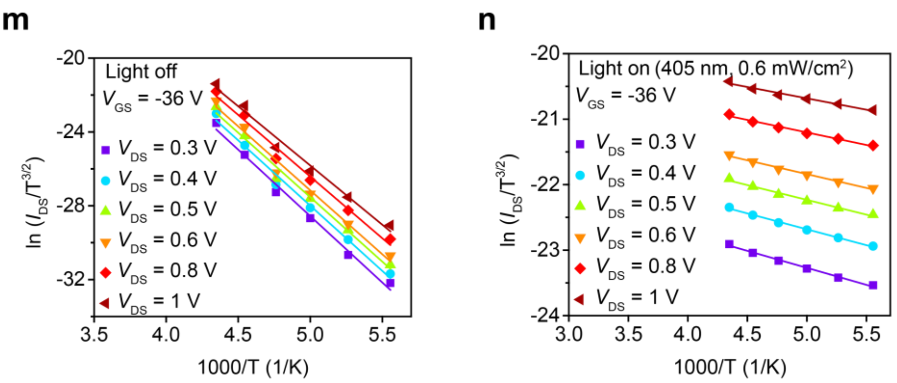

Supplementary Fig. 18 Extraction of Schottky barrier height. **a-f** Transfer characteristics (I_{DS}-V_{GS}) of the device in dark at several temperatures with different V_{DS} from 0.3 V to 1 V. **g-l** I_{DS}-V_{GS} of the device under the light at several temperatures with different V_{DS} from 0.3 V to 1 V. **m** Liner fit of ln($I_{DS}/T^{3/2}$) -1000/T in dark at V_{GS} = −36 V with different V_{DS} from 0.3 V to 1 V. **n** Liner fit of ln($I_{DS}/T^{3/2}$) -1000/T under the light at V_{GS} = −36 V with different V_{DS} from 0.3 V to 1 V.

We measured the temperature dependence of the I_{DS}-V_{GS} traces in a α-MoO_{3-x}/MoS_2/α-MoO_{3-x} device. In fact, there are two junctions in this structure. The Schottky barrier mainly comes from drain side in dark. And under the light, the Schottky barrier height is the sum of source and drain barrier.

The Schottky barrier height ϕ_B can be extracted by the 2D thermionic emission equation[8-11]

$$I_{DS} = AA^*_{2D}T^{3/2}\, exp\left[-\frac{q}{k_B T}\left(\phi_B - \frac{V_{DS}}{n}\right)\right]$$

where A is the contact area of junction, A^*_{2D} is the two-dimensional equivalent Richardson constant, q is the magnitude of the electron charge, k_B is the Boltzmann constant, n is the ideality factor, and V_{DS} is the drain-source bias. As shown in **Supplementary Figs. 18a-l**, the I_{DS}-V_{GS} curves were characterized in dark and under the light (405 nm, 0.6 mW/cm^2) at different V_{DS} and temperatures. The ln($I_{DS}/T^{3/2}$) versus 1000/T was plotted at a low gate voltage region (V_{GS} = −36 V) in dark (**Supplementary Fig. 18m**) and under the light (**Supplementary Fig. 18n**), showing

the ϕ_B is about 0.55 eV in dark and 0.042 eV under a 405 nm light with a power density of 0.6 mW/cm^2 (**Figs. 4d, 4f**). The height of Schottky barrier under the light is much lower than that in the dark, which is consistent with our photo-induced barrier lowering (PIBL) mechanism.

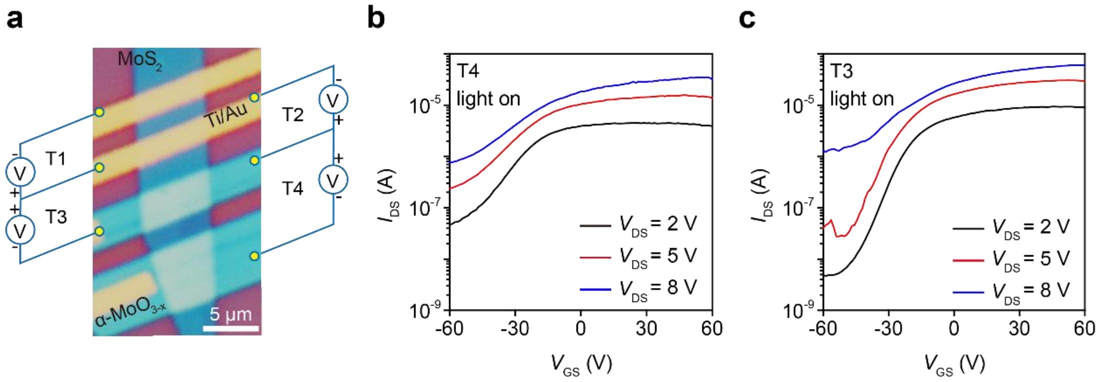

Supplementary Fig. 19 Optoelectrical measurement of T3 and T4. **a** Four MoS_2 phototransistors with difference source (cathode) and drain (anode) electrodes marked T1 (Ti/Au, Ti/Au), T2 (α-MoO_{3-x}, Ti/Au), T3 (Ti/Au, α-MoO_{3-x}), T4 (α-MoO_{3-x}, α-MoO_{3-x}). **b** I_{DS}-V_{GS} curves of T4 under the light at different V_{DS}. **c** I_{DS}-V_{GS} curves of T3 under the light at different V_{DS}.

A control sample clearly highlight our theory. As shown in **Supplementary Fig. 19,** we characterized the I_{DS}-V_{GS} curves for T3 and T4 under the light at different V_{DS}. The photo response of T4 is much better than T3 when the V_{DS} is small ($V_{DS} < 5$ V), due to the feedback effect. However, if the V_{DS} is large enough to smear the barrier at Drain metal contact, the photo response of these two devices is similar (V_{DS} = 8 V). It suggested vdW junction and metal junction are identical in the absence of contact barrier. This phenomenon is inconsistent with the feedback mechanism we previously proposed.

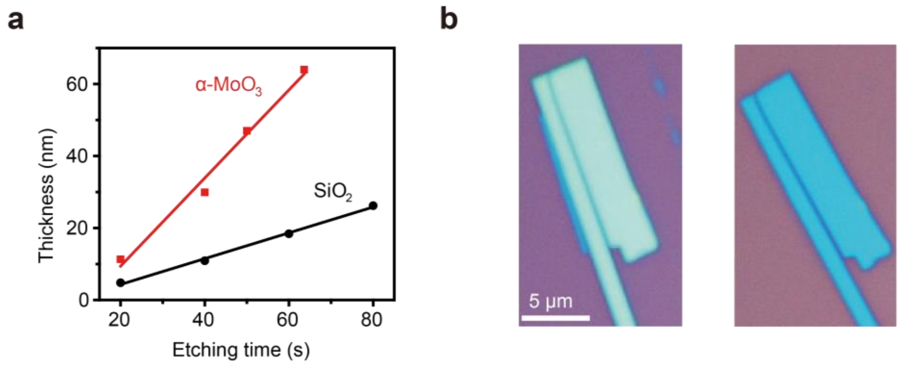

Supplementary Fig. 20 The reactive ion etching (RIE) process of α-MoO_3. **a** Relationship of etching thickness and etching time for α-MoO_3 and SiO_2 by RIE using CHF_3 with a flux rate of 20 sccm and O_2 with a flux rate of 4 sccm, 2.0 Pa pressure and 100 W power. **b** Optical images of an α-MoO_3 flake (left) before and (right) after etching.

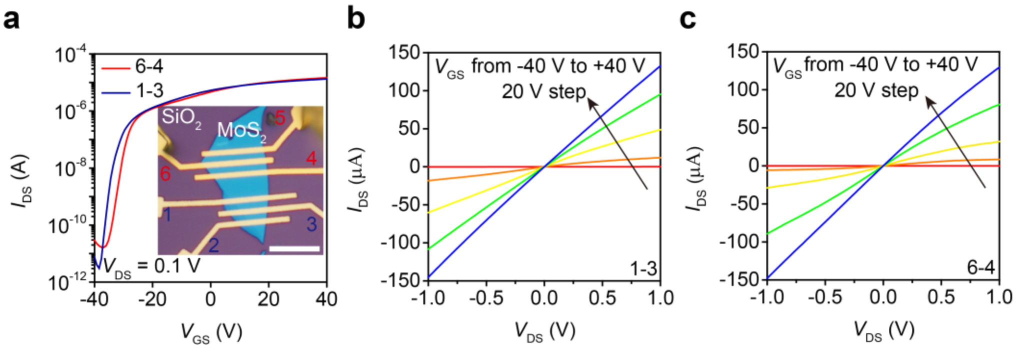

Supplementary Fig. 21 Effect of vacuum annealing on MoS_2. **a** I_{DS}-V_{GS} characteristics of pristine (1-3) and annealed (6-4) MoS_2 based FET at V_{DS} = 0.1 V. The mobilities of the device 1-3 and 6-4 extracted from I_{DS}-V_{GS} curves are 24 cm^2 V^{-1} s^{-1} and 28.5 cm^2 V^{-1} s^{-1}, respectively. Insert: Optical image of the pristine (1, 2, 3) and anealed (4, 5, 6) MoS_2 based FET, fabricated on a 270 nm-thick SiO_2. Scale bar 10 μm. **b** I_{DS}-V_{DS} curves of the device 1-3 with a changing V_{GS} from −40 V to 40 V. **c** I_{DS}-V_{DS} curves of the device 6-4 with a changing V_{GS} from −40 V to 40 V. Ohmic contacts were formed between MoS_2 and Ti/Au electrodes.

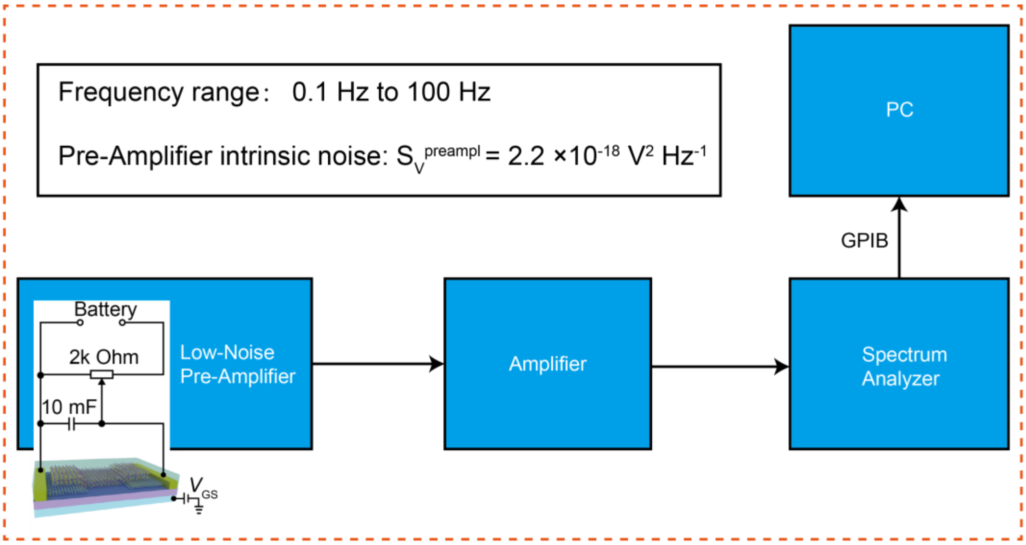

Supplementary Fig. 22 A sketch of the noise measurement system. The sampling frequency of the device ranges from 0.1 Hz to 100 kHz. The GPIB is the abbreviation of general purpose information bus.

A sketch of the noise measurement system (PDANC300L) is shown in **Supplementary Fig. 22.** The system consists of four parts, which are low-noise pre-amplifier, amplifier, spectrum analyzer and personal computer (PC). The sample is supplied with bias voltage (V_{DS}) by a lead-acid battery built into the pre-amplifier. Because the noise coefficient of the system is determined by the first stage, the pre-amplifier is a low noise amplifier. After the two-stage amplification, the noise current is sampled at high speed and analyzed by fast Fourier transform (FFT). Finally, the noise spectrum will be outputted to the PC.

Supplementary References

1. Xiang, D., Han, C., Zhang, J. & Chen, W. Gap states assisted MoO_3 nanobelt photodetector with wide spectrum response. *Sci. Rep.* **4**, 4891 (2014).

2. Zheng, B., Wang, Z., Chen, Y., Zhang, W. & Li, X. Centimeter-sized 2D α-MoO_3 single crystal: growth, Raman anisotropy, and optoelectronic properties. *2D Mater.* **5**, 045011 (2018).

3. Wang, Y. et al. Growth of large-scale, large-size, few-layered alpha-MoO_3 on SiO_2 and its photoresponse mechanism. *ACS Appl. Mater. Interfaces* **9**, 5543-5549 (2017).

4. Dutta, P. & Horn, P. M. Low-frequency fluctuations in solids: 1/f noise. *Rev. Mod. Phys.* **53**, 497–516 (1981).

5. Das, S., Chen, H. Y., Penumatcha, A. V. & Appenzeller, J. High performance multilayer MoS_2 transistors with scandium contacts. *Nano Lett.* **13**, 100-105 (2013).

6. K. Inzani., T. Grande., F. Vullum-Bruer. & S. M. Selbach. A van der Waals density functional study of MoO_3 and its oxygen vacancies. *J. Phys. Chem. C* **120**, 8959-8968 (2016).

7. KC, Santosh. et al. Electronic properties of MoS_2/MoO_x interfaces: implications in tunnel field effect transistors and hole contacts. *Sci. Rep.* **6**, 33562 (2016).

8. Chen, J.-R. et al. Control of Schottky barriers in single layer MoS2 transistors with ferromagnetic contacts. *Nano Lett.* **13**, 3106–3110 (2013).

9. Kraig Andrews, Arthur Bowman, Upendra Rijal, Pai-Yen Chen & Zhixian Zhou.

Improved contacts and device performance in MoS_2 transistors using a 2D semiconductor interlayer. *ACS Nano* **14**, 6232-6241 (2020).

10. Anwar, A., Nabet, B., Culp, J. & Castro, F. Effects of electron confinement on thermionic emission current in a modulation doped heterostructure. *J. Appl. Phys.* **85**, 2663–2666 (1999).

11. Yi, Zhou. et al. Investigating the origin of Fermi level pinning in Ge Schottky junctions using epitaxially grown ultrathin MgO films. *Appl. Phys. Lett.*, **96**, 102103 (2010).
